# Characterization of Turbulent Flow Behind a Transcatheter Aortic Valve in Different Implantation Positions

**DOI:** 10.3389/fcvm.2021.804565

**Published:** 2022-01-13

**Authors:** Leonardo Pietrasanta, Shaokai Zheng, Dario De Marinis, David Hasler, Dominik Obrist

**Affiliations:** ^1^ARTORG Center for Biomedical Engineering Research, University of Bern, Bern, Switzerland; ^2^Dipartimento di Meccanica Matematica e Management, Centro di Eccellenza in Meccanica Computazionale, Politecnico di Bari, Bari, Italy

**Keywords:** TAVI, tomographic particle image velocimetry, turbulence, implantation position, pinwheeling, turbulent spectra

## Abstract

The development of turbulence after transcatheter aortic valve (TAV) implantation may have detrimental effects on the long-term performance and durability of the valves. The characterization of turbulent flow generated after TAV implantation can provide fundamental insights to enhance implantation techniques. A self-expandable TAV was tested in a pulse replicator and the three-dimensional flow field was extracted by means of tomographic particle image velocimetry. The valve was fixed inside a silicone phantom mimicking the aortic root and the flow field was studied for two different supra-annular axial positions at peak systole. Fluctuating velocities and turbulent kinetic energy were compared between the two implantations. Velocity spectra were derived at different spatial positions in the turbulent wakes to characterize the turbulent flow. The valve presented similar overall flow topology but approximately 8% higher turbulent intensity in the lower implantation. In this configuration, axial views of the valve revealed smaller opening area and more corrugated leaflets during systole, as well as more accentuated pinwheeling during diastole. The difference arose from a lower degree of expansion of the TAV's stent inside the aortic lumen. These results suggest that the degree of expansion of the TAV *in-situ* is related to the onset of turbulence and that a smaller and less regular opening area might introduce flow instabilities that could be detrimental for the long-term performance of the valve. The present study highlights how implantation mismatches may affect the structure and intensity of the turbulent flow in the aortic root.

## 1. Introduction

Aortic stenosis is the primary valvular disease with increasing prevalence due to an aging population (1, 2). Untreated severe symptomatic aortic stenosis has a fatal prognosis with a mortality rate up to 50% in the first year and higher than 90% after 5 years (3). Transcatheter aortic valve implantation (TAVI) is an established treatment for high-risk patients which are not eligible for surgery. Recently, guidelines were updated and TAVI was introduced as a possible treatment for a larger population which include intermediate-risk patients (4–7). TAVI demonstrated significant survival benefit in the short-term follow ups across the spectrum of intermediate and high-risk patients when compared to the classical surgical valve replacement (SVR) (8, 9). More recent studies based on longer follow ups also showed promising improvements in patients' quality of life (10) and health status (11). Current debates focus on the expansion of TAVI to patients with low operative risk which have a longer life expectancy (12). Consequently, long-term structural device integrity is an important goal for transcatheter aortic valve (TAV) design. Turbulence might be considered as one of the detrimental phenomena that could affect the long-term performance of TAV and lead over time to clinically adverse events. Turbulent flow is not only directly related to increased transvalvular pressure gradients (13) but also to structural valve deterioration (SVD) caused by excessive stress loads on the valve's leaflets (14, 15). Turbulence could also lead to endothelial lesions and aorta remodeling due to unphysiological wall shear stress (16), as well as thrombus formation due to the activation of platelets (17, 18). The high degree of freedom present during implantation makes TAVs particularly prone to the development of turbulent flows due to potential geometrical mismatches. Even though guidelines are given to practitioners, the final geometrical configuration of the TAV is highly patient-specific due to the specific anatomy and different degree of calcification of the aortic root (19). Different implantation configurations may lead to different degree of turbulent flow. Despite the important role of turbulence there is a lack of understanding of this phenomenon. In theory, direct numerical simulations can resolve the smallest scales of turbulent flow structures providing the information for a full characterization of turbulence. However, the available numerical findings focus on surgical valves (SVs) (13, 20–22) and the ones on TAV are limited and need to be cross-validated with experimental results (23–26). One of the first fluid structure interaction (FSI) simulations on TAV was performed by Mao et al. (23) who investigated the leaflets' kinematics and stress distribution on a simplified TAV model without considering the stent geometry. A comparison between FSI simulation and *in-vitro* measurements of a self-expandable TAV was obtained for an idealized experimental condition by Wu et al. (24), yet the comparison was limited to the quantitative analysis of the TAV's opening area and did not investigate the flow field. A FSI simulation of a CoreValve (*Medtronic Inc, Minneapolis, Minnesota, USA*) in a more realistic geometry was performed by Kandail et al. (25), who considered an anatomically correct aortic arch but focused on the flow patterns in the coronary arteries. More recently, Basri et al. (26) studied the impact of paravalvular leakage using the FSI approach. None of the mentioned works investigated the role of turbulence generated after TAVI. On the other hand, experimental studies are often limited by the measurement technology which often lack sufficient resolution and only allow for one or two-dimensional analysis of turbulent flow (27–30). Therefore, further investigations are needed to better characterize the turbulent three-dimensional flow and its potential effect on the long-term performance of TAVs. To the best of our knowledge, this study is the first experimental investigation that attempts to characterize the turbulent wake generated by a TAV with tomographic particle image velocimetry (Tomo-PIV).

TAVs may block the electrical signal in the atrio-ventricular node or in the left bundle branch due to balloon dilatation or prosthesis implantation. Conduction abnormalities have to be treated with the implantation of pacemakers. Furthermore, TAVs may impair the coronary flow due the displacement of the calcified native cusp over the coronary ostium or due to the TAV's skirt which prevent the blood flow from entering the sinus of Valsalva. As suggested by Yerasi et al. (31) and Ribeiro et al. (32) a higher TAV implantation is related to a higher risk for coronary obstruction. From this perspective, a lower implantation should be preferred. However, Van Rosendael et al. (33) and Mauri et al. (34) reported higher pacemaker implant rate in lower implantations. Indipendent of this important discussion, we will discuss here the effect of the longitudinal positioning and the resulting turbulence for two different implantation heights.

## 2. Methods

*In-vitro* measurements of the three-dimensional flow field in the aortic root generated by a TAV were performed in a validated pulse replicator with a multi-view imaging system for Tomo-PIV (35). The experimental apparatus already used by the authors for studies of surgical heart valve (36) was further adapted for TAV testing.

### 2.1. Pulse Replicator

**Figure 1:**
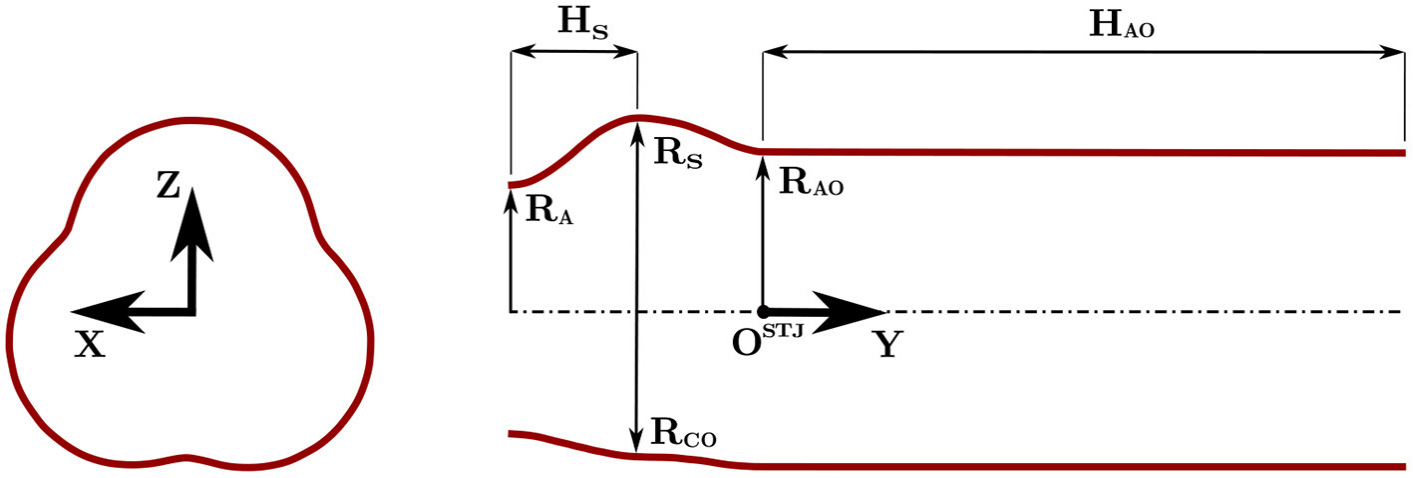
Inner lumen geometry of the silicone phantom mimicking the aortic root with reference to the main geometrical parameters of Table 1.

**Figure 2:**
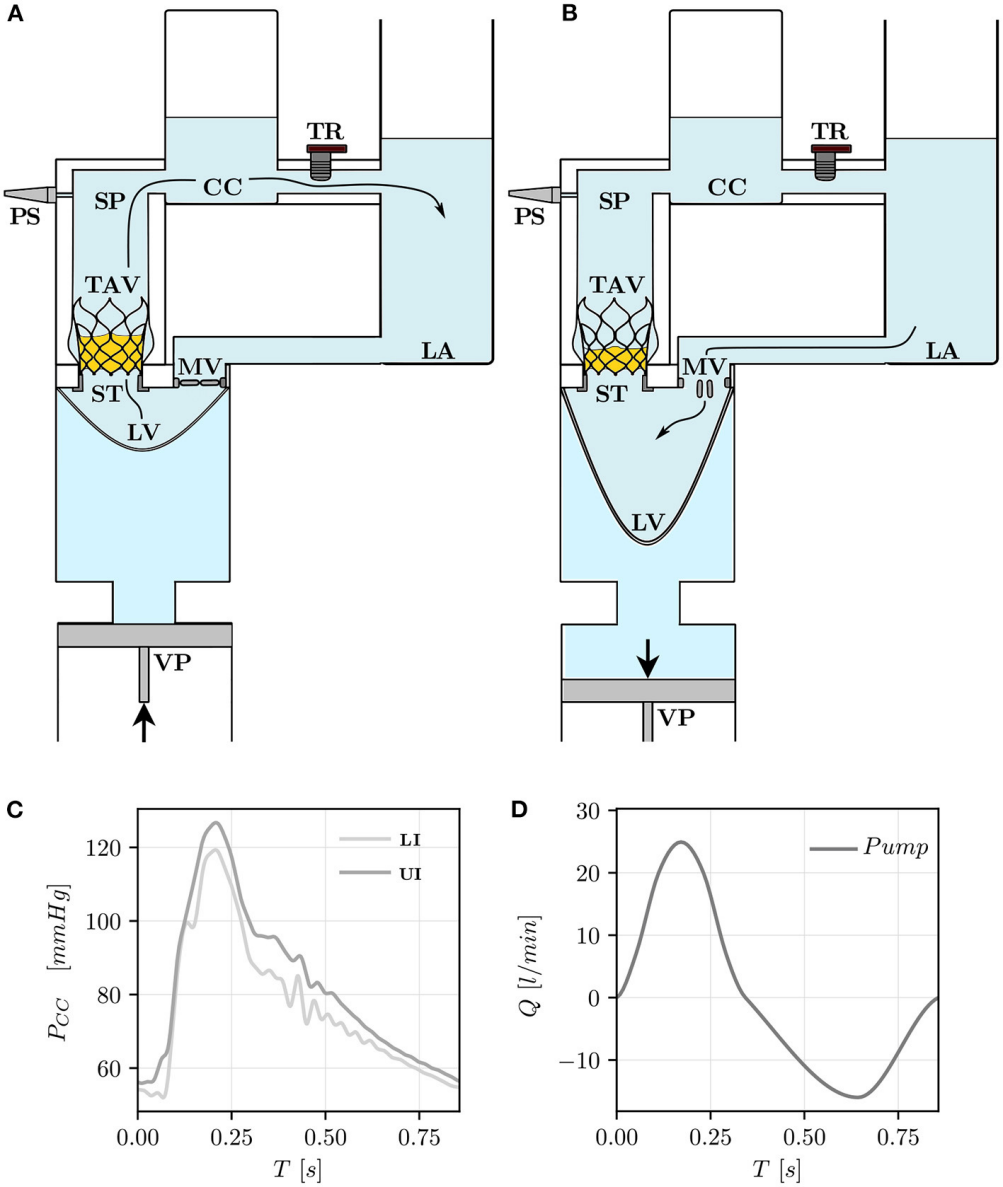
Pulse replicator and its elements during the systolic phase **(A)** and the diastolic phase **(B)**. The pulse replicator is a hydraulic flow loop consisting of the volumetric pump (VP), the left ventricle (LV), the transcatheter aortic valve (TAV), the stopper (ST), the compliance chamber (CC), the tunable resistor (TR), the left atrium (LA), the mitral valve (MV), and the pressure sensors (PSs). Pressure signals in the CC (*P*_*CC*_) are shown in **(C)** for the UI and the LI. The flow rate (*Q*) prescribed at the outlet of the VP (*Pump*) is shown in **(D)**.

The tested TAV was crimped at 0°*C* and released in a semi-rigid silicone phantom (SP) with an idealized aortic root geometry (Figure 1) at 37°*C*. The silicone phantom was characterized by an aortic annulus diameter *D*_a_ = 23 *mm*, three hemispherical bulges mimicking the sinuses of Valsalva and a straight ascending aorta with a diameter of 29 *mm*. More details on the aortic root phantom can be found in Table 1. The silicone phantom and the TAV were inserted in the test section of a pulse replicator which reproduced physiological pulsatile flow using a computer-controlled volumetric pump (VP). The motion of the VP's piston was tuned to replicate physiological flow profiles characterized by 1/3 of systolic phase and 2/3 of diastolic phase. A schematic of the operation of the pulse replicator is shown in Figure 2A for the systolic phase and in Figure 2B for the diastolic phase. During systole, a silicone left ventricle (LV) was compressed by the forward motion of the VP's piston and the fluid was directed toward the test section. After passing through the test section, the fluid reached a sealed compliance chamber (CC) and a tunable resistor (TR) mimicking the arterial compliance and the peripheral resistance of the systemic circulation, respectively. Finally, the fluid was collected in an open chamber exposed to atmospheric pressure simulating the left atrium (LA). During diastole the silicone ventricle was expanded to its original relaxed state by the backward motion of the VP's piston and the fluid was returned from the open chamber to the silicone ventricle. To mimic the function of the mitral valve a mechanical bi-leaflet valve (MV) was installed between the open chamber and the silicone ventricle. Pressure sensors (PSs) were positioned in the left ventricle and in the compliance chamber to estimate the left ventricular pressure and the aortic pressure. A blood analog test fluid with a density of ρ_f_ = 1, 200 *kg*/*m*^3^ and a kinematic viscosity ν = 4.7 *mm*^2^/*s* at 22°*C* was used. The test fluid consisted of 49.4 *w%* water, 34 *w%* glycerol (*Dr. Grogg Chemie AG, Deisswil, Switzerland*) and 16.6 *w%* sodium chloride (*Sigma-Aldrich Corporation, St. Louis, MO, USA*) by weight. The test fluid had a refraction index similar to the one of silicone to reduce distortion during image acquisition.

**Table 1:** Relevant geometrical parameters of the aortic root silicone phantom.

**Parameter**	**Value **[*mm*]****	**Description**
H_S_	11.6	Sinus half-height
H_AO_	58.9	Ascending aorta height
R_A_	11.5	Aortic annulus radius
R_S_	17.6	Sinus radius
R_CO_	13.1	Commissure radius
R_AO_	14.5	Ascending aorta radius

### 2.2. TAV Model

**Figure 3:**
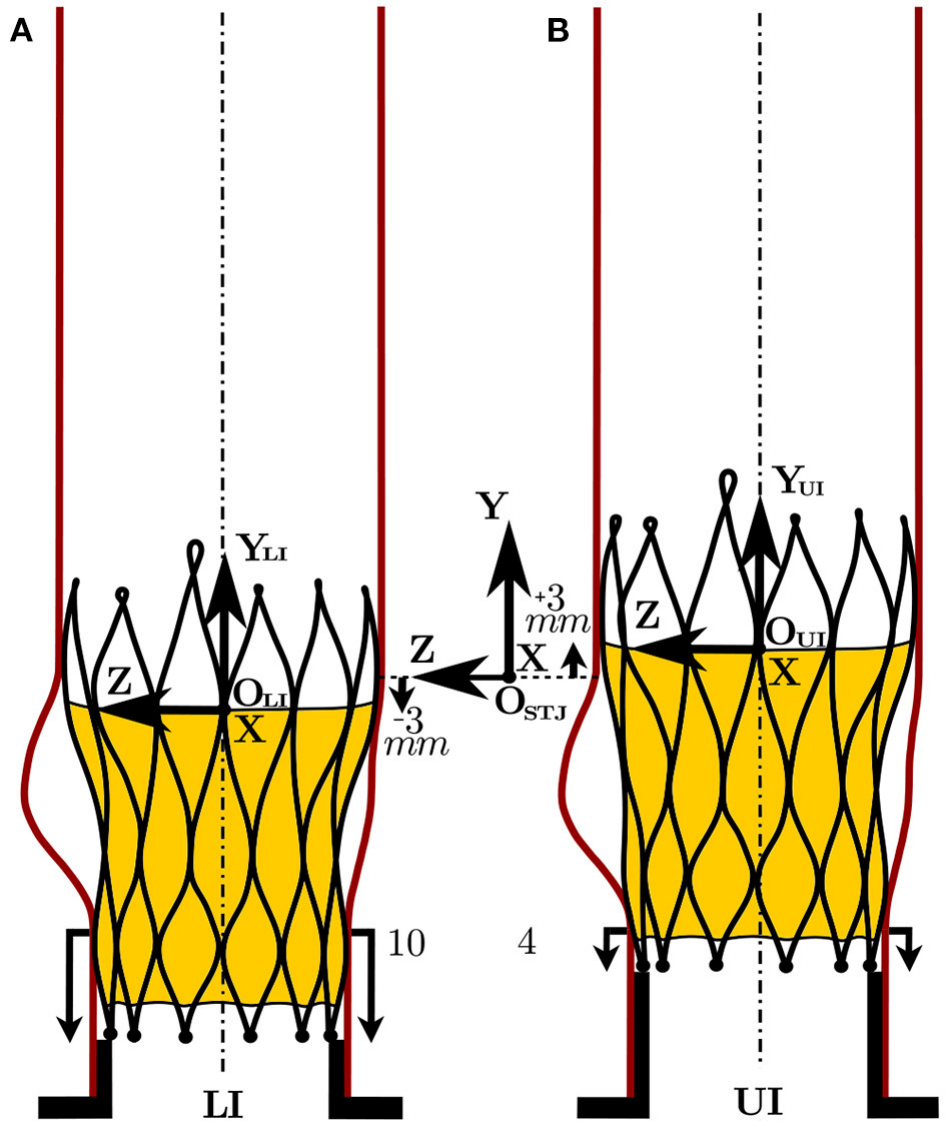
TAV axial positioning in the LI **(A)** and in the UI **(B)** and the relative positioning of the LI (O^LI^) and the UI (O^UI^) reference systems with respect to the measurement reference system (O^STJ^).

The TAV featured three leaflets made of porcine pericardium sutured onto a self-expandable Nitinol stent frame. The nominal valve prosthesis size was 29 *mm* which is suitable for implantation in aortic roots with an aortic annulus diameter *D* = 23 − 26 *mm* according to manufacturer's recommendation. To ensure the correct axial position, the TAV was positioned over a stopper (ST). The stopper was designed as a tubular structure with the same wall thickness as the valve's stent struts. The lower extremities of the TAV's struts touched the upper end of the stopper avoiding migration toward the ventricle. The TAV was oriented in a “native configuration” with the leaflets facing the sinuses of Valsalva. The axial positioning, or implantation height (IH), was controlled through the stopper such that the lower extremities of the TAV's stent were 10 *mm* distal to the aortic annulus of the silicone phantom (“lower implantation,” LI, Figure 3A). A second configuration (“upper implantation,” UI, Figure 3B) 4 *mm* distal to the annulus was considered to span the full range of IH suggested by the manufacturer.

A high-speed camera (*Photron AX 100, Tokyo, Japan*) was used to capture axial top view images of the tested TAV. Valve kinematics was assessed by direct inspection of the axial high speed recordings. The geometric orifice area (*GOA*) was defined by a contour line along the TAV's leaflet tips and was manually selected from the axial images. The projected orifice area (*POA*) was defined as the open area seen by the axial camera and was estimated with the same procedure. The presented results are mean values over five consecutive pulses. The pinwheeling index (*PI*) of Midha et al. (37) was used to quantify localized bending of TAV's leaflets along the coaptation edge during diastole:


(1) $$\begin{array}{l} PI=\frac{L_{actual}-L_{ideal}}{L_{ideal}} \end{array}$$

where *L*_*actual*_ is the length of the coaptation edge and *L*_*ideal*_ is the distance between the commisures and the center of the valve. The presented results are mean values of the three coaptation edges. The *PI* of each of the three coaptation edges was evaluated as an average over five consecutive pulses.

### 2.3. Tomo-PIV Measurement System

Two 8M 12 bit CCD Digital Cameras (*Imager LX, LaVision, Gttingen, Germany*) and a set of mirrors were used to image the aortic root flow domain. The field of view of each camera was split into two images such that four different viewing angles were captured simultaneously with only two cameras. The cameras were equipped with prime lenses with a focal length of *f* = 100 *mm* and a maximum aperture of F2.8 (*Kenko Tokina, Tokyo, Japan*). Each of the four recorded images had a resolution of 1656 × 2488 pixels. The pixel size was Δ_*px*_ = 5.5 μ*m*, while the magnification factor *M* resulting from the focal length *f* and the object distance *S* = 0.7 *m* was *M* = *f*/(*S* − *f*) = 0.167. The test fluid was seeded with fluorescent PMMA micro particles of mean diameter *D*_p_ = 35 μ*m* (density ρ_p_ = 1180 *kg*/*m*^3^) with Rhodamin B coating (*Microparticles GmbH, Berlin, Germany*) that has an excitation peak at wavelength λ = 560 *nm* and emission peak at wavelength λ = 584 *nm*. A dual cavity Nd:YAG laser (*Nano L 200-15 PIV, Litron Systems Ltd, Rugby, UK*) was used to excite the fluorescent particles at λ = 532 *nm* with a power of 235 *mJ*/*pulse*. The time width of the laser was set to 7 *ns* and the interframe time between consecutive laser pulses was set to 200 μ*s*. A low-pass filter (570 *nm* cut-off wavelength) in front of the cameras ensured that only the emitted light from the particles was collected by the camera sensors. The side views of the CCD cameras where used to capture the light emitted by the fluorescent particles, as well as to measure the expansion rate of the TAV in the silicon phantom.

The software *DaVis 8.4* (*LaVision GmbH, Gttingen, Germany*) was used for camera calibration, raw image pre-processing, volume reconstruction and for the computation of the 3D instantaneous velocity vector fields with a 3D cross-correlation algorithm (tomographic particle image velocimetry, Tomo-PIV). The resulting 3D instantaneous velocity vector field


(2) $$\begin{array}{l} v\left(x,t\right)\text{ where }v=\left[v_{x},v_{y},v_{z}\right]\text{ and }x=\left[x,y,z\right] \end{array}$$

was defined on a Cartesian voxel grid. As shown in Figure 3, the origin O_STJ_ of the Cartesian coordinate system was located on the centerline of the aortic root at the height of the sinotubular junction (STJ). Because the TAV leaflets' trailing edge during systole was 3 *mm* below O_STJ_ in the LI and 3 *mm* above O_STJ_ in the UI, two additional coordinates systems were defined to have a correspondence with respect to the valve's trailing edge. Thus, a coordinate system O_LI_ for the LI was placed 3 *mm* below O_STJ_ while a coordinate system O_UI_ for the UI was positioned 3 *mm* above O_STJ_:

O_LI_ reference system: ***x***_LI_ = [*x, y*_LI_, *z*] where *y*_LI_ = *y* − 3 *mm*;

O_UI_ reference system: ***x***_UI_ = [*x, y*_UI_, *z*] where *y*_UI_ = *y* + 3 *mm*.

**Figure 4:**
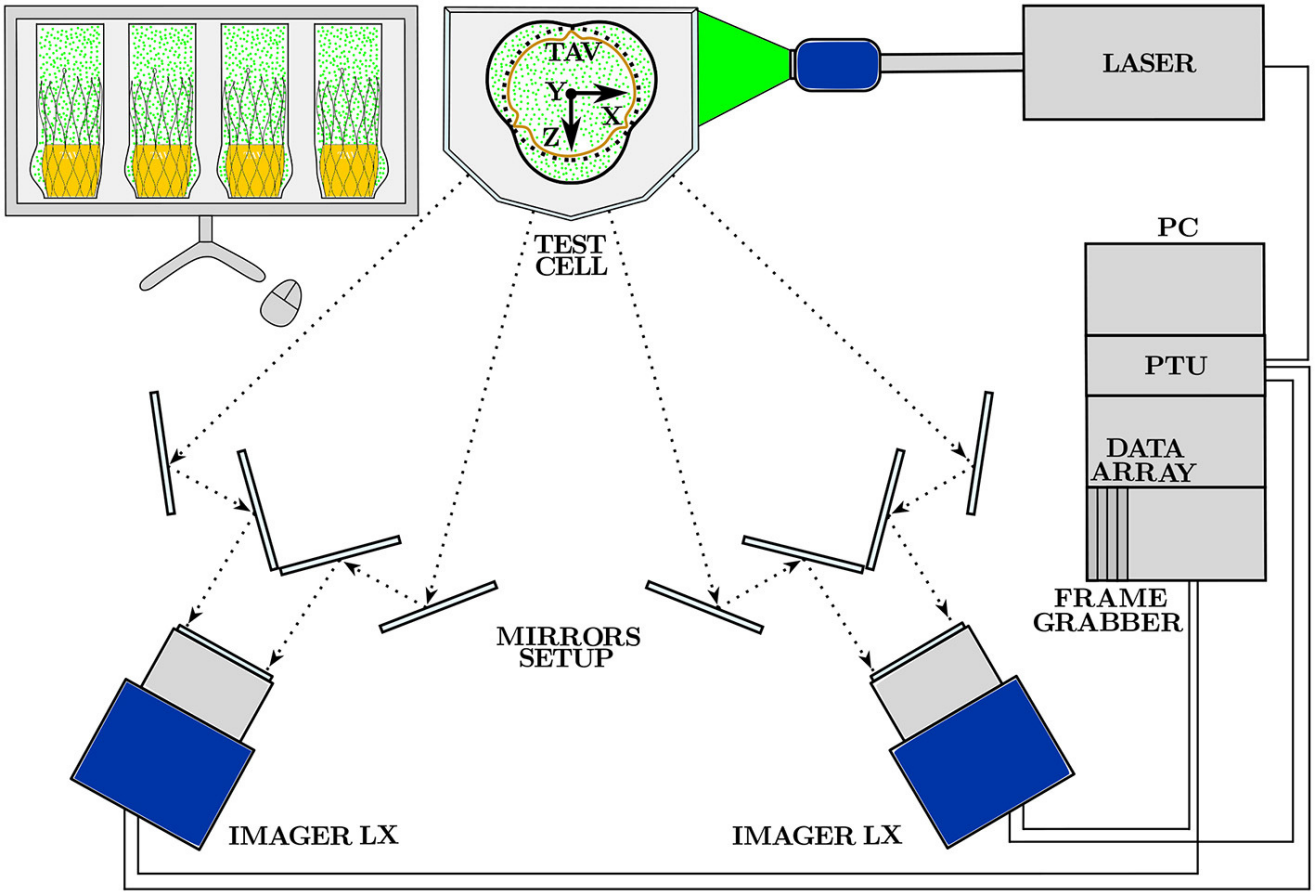
Scheme of the Tomo-PIV hardware together with the measurement reference system. The main components for image acquisition are: two CCD Digital Camera, a set of mirrors and a dual cavity Nd:YAG laser with optics for volumetric illumination. Image storage and processing is performed with the software *DaVis 8.4* installed on the PC workstation.

A schematic of the main components of the hardware for image acquisition and processing of the experimental apparatus with reference to the measurement Cartesian reference system can be found in Figure 4.

The experimental resolution δ was the same in all spatial directions (δ_*x*_ = δ_*y*_ = δ_*z*_) and was estimated from the voxel size (Δ_*vx*_ = Δ_*px*_/*M*) and from the dimension of the spherical interrogation volume and overlap set for the last step of the 3D cross-correlation routine. The interrogation volume was set to 48 *voxels*, which is equivalent to 1.58 *mm*. With 75% correlation overlap, the final resolution of the velocity field was:


(3) $$\begin{array}{l} \delta =48\times \left(1-0.75\right)\times \Delta_{vx}\approx 0.43\;mm. \end{array}$$

Further details on Tomo-PIV are given in [1].

### 2.4. Experimental Protocol

The experiments were performed at room temperature (≈ 22°C) with a heart rate of 70 *bpm* which led to a heart cycle of period *T* = 0.86 *s*. Figures 2C,D show the hemodynamics boundary conditions (averaged over five consecutive pulses) under which the TAV operated during the measurements. A cardiac output of approximately 5 *l*/*min* with *Q*_max_ ≈ 25 *l*/*min* resulted from the prescribed pump settings. The pressure in the compliance chamber (CC) ranged between 50 and 130 *mmHg* for both implantation configurations. The Reynolds number (*Re*) and the Womersley number (*Wo*) at peak flow were:


(4) $$\begin{array}{l} Re=\frac{4Q_{\text{max}}}{D_{a}\pi \nu }\approx 5000\text{ and }Wo=R_{a}\sqrt{\frac{2\pi }{T\nu }}\approx 14 \end{array}$$

where *D*_*a*_ = 23 *mm* and *R*_*a*_ = *D*_*a*_/2 = 11.5 *mm* are the diameter and radius of the aortic annulus, respectively.

At this Reynolds number, turbulent flow is expected. *N* acquisitions of the instantaneous flow field at different phases (ϕ) of the cardiac cycle were performed with a phase-locked approach such that every acquisition was obtained at time *t* = *t*_ϕ_ + *nT* where *n* = 1, 2, …, *N* indicates the repetition number.

The mean velocities $\bar{v}\left(x\right)$ were computed by *phase-averaging* the instantaneous velocity field such that:


(5) $$\begin{array}{l} \bar{v}\left(x\right)=\frac{1}{N}\overset{N}{\underset{n=1}{\sum }}v\left(x,t_{\phi }+nT\right) \end{array}$$

where $\bar{\cdot }$ denotes the *phase-averaging* operation over the *N* repetitions of the experiment. At peak flow phase (*t*_peak_ = 0.17 *s*) *N* = 64 realizations were acquired; *N* = 24 acquisitions were also performed at *t*_ϕ_ between 0.05 − 0.17 *s* with Δ*t*_ϕ_ = 0.01 *s*, and between 0.20 − 0.40 *s* with an interval Δ*t*_ϕ_ = 0.05 *s*.

### 2.5. Flow Field Analysis

The overall topology of the flow field in the different phases of the heart cycle was assessed by studying the mean streamwise velocity component ${\bar{v}}_{y}$ and the mean velocity magnitude:


(6) $$\begin{array}{l} |\bar{v}|=\sqrt{{\bar{v}}_{x}^{2}+{\bar{v}}_{y}^{2}+{\bar{v}}_{z}^{2}}. \end{array}$$

From the velocity fields ***v***, the velocity fluctuations fields ***v***′ were computed according to the Reynolds decomposition (38):


(7) $$\begin{array}{l} v=\bar{v}+v^{\prime } \end{array}$$

where ′ indicates the velocity fluctuation. Note that the fluctuations ***v***′ may include turbulent fluctuations, as well as pulse-to-pulse fluctuations. The standard deviation σ_*i*_ of the velocity fluctuations $v_{i}^{\prime }$ was defined as:


(8) $$\begin{array}{l} \sigma_{i}=\sqrt{\bar{v_{i}^{\prime 2}}}\text{ with }i=x,y,z; \end{array}$$

while the root mean square of the velocity fluctuations was computed as:


(9) $$\begin{array}{l} v_{rms}^{\prime }=\sqrt{\bar{v_{x}^{\prime 2}}+\bar{v_{y}^{\prime 2}}+\bar{v_{z}^{\prime 2}}}. \end{array}$$

At peak flow, sufficient convergence of the $v_{rms}^{\prime }\left(x\right)$ was obtained on the centerline of the flow domain (***x*** = [0, *y*, 0]) already with *N* = 50 repetitions (see Supplementary Material).

The mean kinetic energy density *mke*(***x***) [*J*/*kg*] and the turbulent kinetic energy density *tke*(***x***) [*J*/*kg*] were defined as:


(10) $$\begin{array}{l} mke=\frac{1}{2}\left({\bar{v}}_{x}^{2}+{\bar{v}}_{y}^{2}+{\bar{v}}_{z}^{2}\right)=\frac{1}{2}\;|\bar{v}{|}^{2} \end{array}$$

(11) $$\begin{array}{l} tke=\frac{1}{2}\left(\bar{v_{x}^{\prime 2}}+\bar{v_{y}^{\prime 2}}+\bar{v_{z}^{\prime 2}}\right)=\frac{1}{2}\;v_{rms}^{\prime 2}. \end{array}$$

Integration over the volume V yielded the total mean kinetic energy *MKE* [*J*] and the total turbulent kinetic energy *TKE* [*J*]:


(12) $$\begin{array}{l} MKE=\rho \int_{\text{V}}mke\;d\text{v} \end{array}$$

(13) $$\begin{array}{l} TKE=\rho \int_{\text{V}}tke\;d\text{v}. \end{array}$$

The turbulence intensity (*TI* [%]) was estimated as the ratio of *TKE*/*MKE*.

A pointwise auto-covariance Γ_*ii*_(***l***) between fluctuations at different locations separated by a distance ***l*** was computed according to:


(14) $$\begin{array}{l} \Gamma_{ii}\left(l;x,t_{\phi }\right)=\bar{v_{i}^{\prime }\left(x+l,t_{\phi }\right)v_{i}^{\prime }\left(x,t_{\phi }\right)}\text{ with }l=\left[l_{x},l_{y},l_{z}\right]. \end{array}$$

The turbulent velocity spectrum *E*_*ii*_(***k***) was computed according to the Wiener-Khinchin theorem:


(15) $$\begin{array}{l} E_{ii}\left(k;x,t_{\phi }\right)=\frac{1}{2\pi }\int_{-\infty }^{+\infty }e^{-ikl}\Gamma_{ii}\left(l;x,t_{\phi }\right)dl\text{ with }k=\left[k_{x},k_{y},k_{z}\right] \end{array}$$

where ***k*** represents the wavenumber.

The energy dissipation rate ϵ was calculated from the rate of strain tensor of the velocity fluctuations as:


(16) $$\begin{array}{l} \epsilon =2\nu \bar{\underset{ij}{\sum }S_{ij}^{2}} \end{array}$$

where *S*_*ij*_ is the symmetric component of the velocity fluctuation gradient tensor:


(17) $$\begin{array}{l} S_{ij}=\frac{1}{2}\left(\frac{\partial v_{i}^{\prime }}{\partial x_{j}}+\frac{\partial v_{j}^{\prime }}{\partial x_{i}}\right)\text{ with }i=x,y,z\text{ and }j=x,y,z. \end{array}$$

Local isotropy and homogeneity was assumed to compute the Kolmogorov length scale η from the energy dissipation rate ϵ as:


(18) $$\begin{array}{l} \eta ={\left(\frac{\nu^{3}}{\epsilon }\right)}^{\frac{1}{4}}. \end{array}$$

Finally, the integral length scale *I*_*ii*_ was estimated as:


(19) $$\begin{array}{l} I_{ii}\left(l\right)=\int_{L}R_{ii}\left(l\right)dl \end{array}$$

where *L* represents the maximum correlation distance along direction ***l*** and *R*_*ii*_(***l***) is the pointwise auto-covariance normalized with the standard deviations of the velocity fluctuations σ_*i*_(***x***):


(20) $$\begin{array}{l} R_{ii}\left(l;x,t_{\phi }\right)=\frac{\Gamma_{ii}\left(l;x,t_{\phi }\right)}{\sigma_{i}\left(x+l\right)\sigma_{i}\left(x\right)}=\frac{\bar{v_{i}^{\prime }\left(x+l\right)v_{i}^{\prime }\left(x\right)}}{\sqrt{\bar{v_{i}^{\prime }{\left(x+l\right)}^{2}}}\sqrt{\bar{v_{i}^{\prime }{\left(x\right)}^{2}}}}. \end{array}$$

## 3. Results

### 3.1. TAV Characteristics

**Figure 5:**
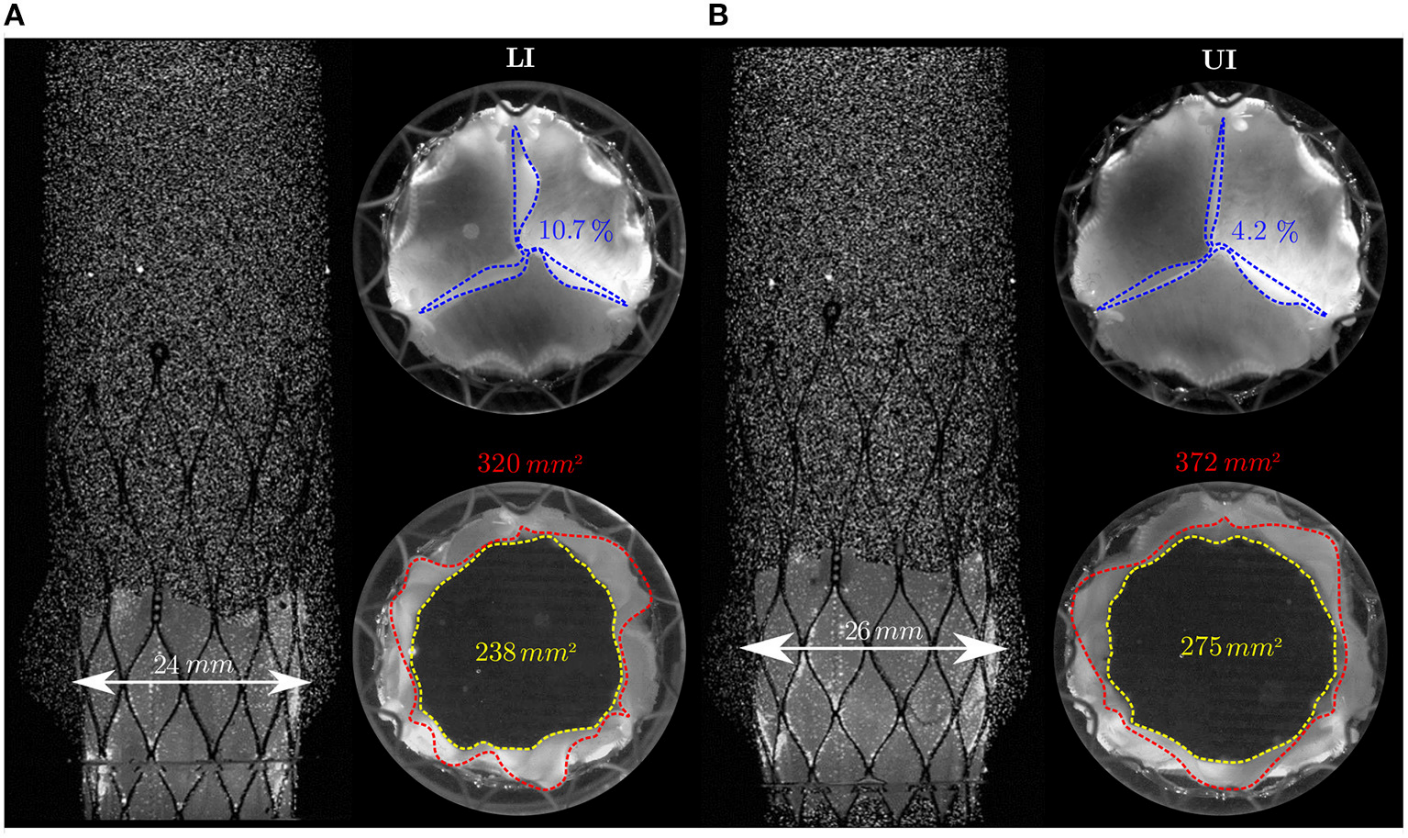
Side and axial views of the TAV in the LI **(A)** and in UI **(B)**. The top views show the TAV at peak flow (*t*_ϕ_ = 0.17 *s*) and at the end of the diastole (*t*_ϕ_ = 0.86 *s*). The side view shows the TAV at peak flow. Red dashed lines highlight the TAV's leaflets tips during systole (*GOA*) while blue lines highlight the TAV's leaflets tips during diastole (*PI*). The *POA* is highlighted by yellow dashed lines. The white arrows denote the TAV's degree of expansion in the aortic root silicone phantom at the level of the sinus portion.

Valve opening and closing times were estimated as an average over five consecutive pulses from direct inspection of the recorded axial view. In the LI, the valve started opening at *t*_ϕ_ = 0.06 *s* with respect to the beginning of the forward motion of the piston pump and was fully open at *t*_ϕ_ = 0.11 *s*. Valve closure started at *t*_ϕ_ = 0.24 *s* and was completed at *t*_ϕ_ = 0.41 *s*. The resulting opening and closing time was 0.05 *s* and 0.17 *s*, respectively. In the UI the valve opened slightly earlier at *t*_ϕ_ = 0.05 *s* but showed otherwise the same opening and closing kinematics. The IH had an effect on the TAV expansion (Figure 5). At the level of the sinus of Valsalva, the TAV's stent struts in the LI expanded to a diameter of 24 *mm*, whereas they expanded to 26 *mm* in the UI. Consequently, the *GOA* in the UI ($GOA_{\text{UI}}=372\;mm^{2}$) was 14% larger than the the *GOA* in the LI ($GOA_{\text{LI}}=320\;mm^{2}$). A difference of 13.5% was observed from the POAs as well ($POA_{\text{UI}}=275\;mm^{2},POA_{\text{LI}}=238\;mm^{2}$). The different opening areas affected the Reynolds number of the flow fields downstream the TAV in the two implantation configuration. Local Reynolds numbers were estimated close to the leaflets' leading edge at peak systole for both IH as:


(21) $$\begin{array}{l} Re_{\text{LI}}=\frac{4Q_{\text{max}}}{D_{\text{LI}}^{GOA}\pi \nu }\approx 5600\;Re_{\text{UI}}=\frac{4Q_{\text{max}}}{D_{\text{UI}}^{GOA}\pi \nu }\approx 5200 \end{array}$$

where $D_{\text{LI}}^{GOA}=20.2\;mm$ and $D_{\text{UI}}^{GOA}=21.8\;mm$ are diameters computed from circular openings with equivalent areas of *GOA*_LI_ and *GOA*_UI_, respectively. Moreover, the TAV in LI showed corrugated and unevenly opened leaflets during systole and accentuated pinwheeling (*PI*_LI_ = 10.7%). Whereas, in UI, it had a more regular orifice area and less pinwheeling (*PI*_UI_ = 4.2 %). The reported characteristics are summarized in Table 2.

**Table 2:** Geometrical features of the expanded TAV's leaflet (*POA*, *GOA*, *PI*), estimated Reynolds number (*Re*) at leaflet tips and integral quantities in the front half of the flow domain (*MKE*, *TKE* and *TI* = *TKE*/*MKE* for *z* > 0).

	***POA* [*mm*^2^]**	***GOA* [*mm*^2^]**	***PI* [%]**	**Re**	***MKE* [*mJ*]**	***TKE* [*mJ*]**	***TI* [%]**
LI	238	320	10.7	5600	5.1	1.0	19.6
UI	275	372	4.4	5200	4.4	0.5	11.4

### 3.2. Flow Characteristics

The streamwise velocity component ${\bar{v}}_{y}$, the velocity fluctuations $v_{rms}^{\prime }$ , the turbulent kinetic energy density *tke*, the turbulent velocity spectra together with the Kolmogorov and the integral scales are presented in this section for the peak flow phase (*t*_ϕ_ = 0.17 *s*).

#### 3.2.1. Mean Streamwise Velocity

**Figure 6:**
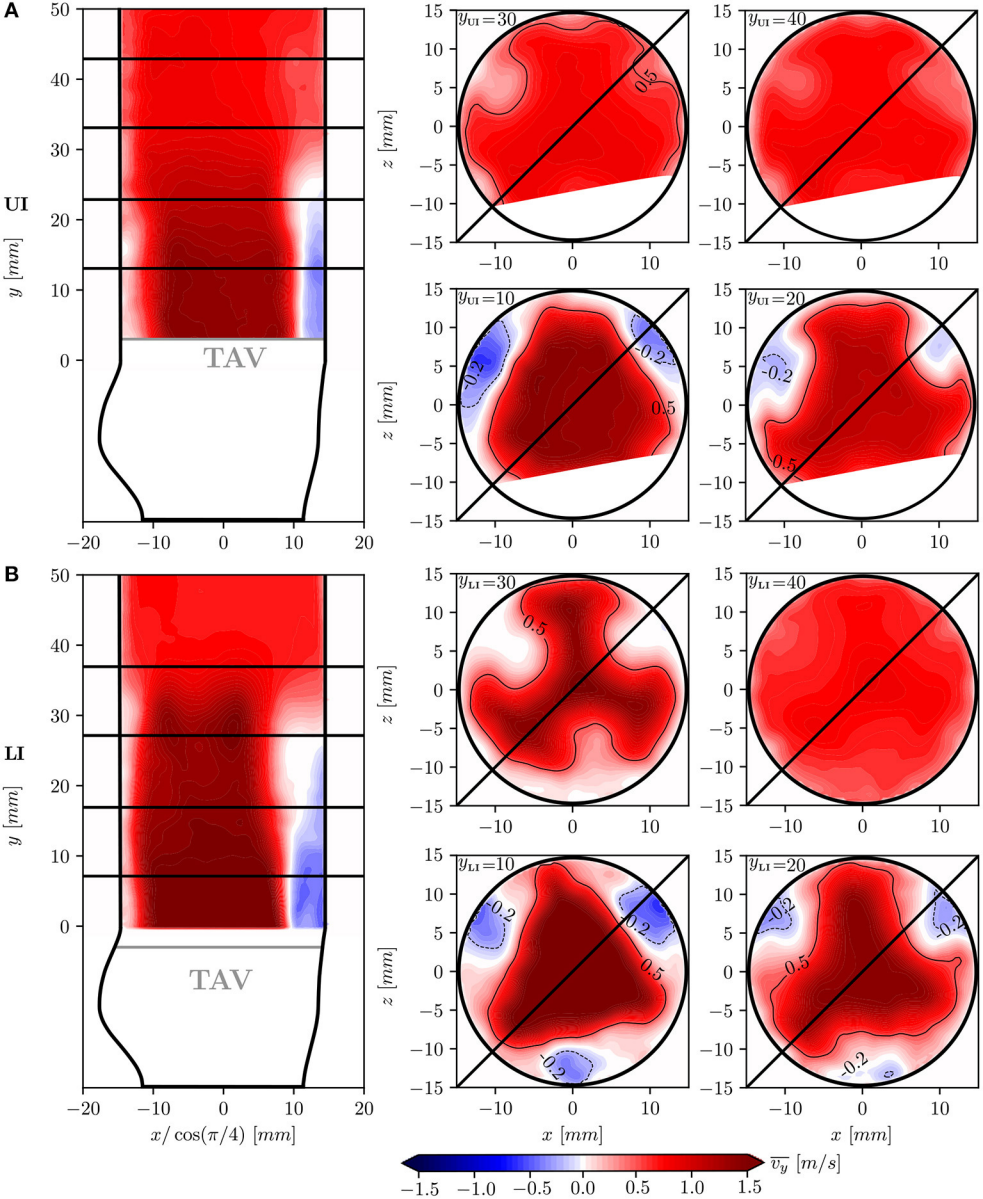
Mean streawise velocity ${\bar{v}}_{y}$ in different cross-sections of the flow-domain: longitudinal plane at 45° together with transversal planes at *y*_LI_ = *y*_UI_ = 10, 20, 30, 40 *mm* for the UI **(A)** and for the LI **(B)**. The core jet is highlighted by contour lines which include ${\bar{v}}_{y}$ values higher than 0.5 *m*/*s*. High $v_{rms}^{\prime }$ values were found close to the aortic wall for the UI due to measurement artifacts. This portion of the flow domain was therefore discarded for subsequent analysis.

**Figure 7:**
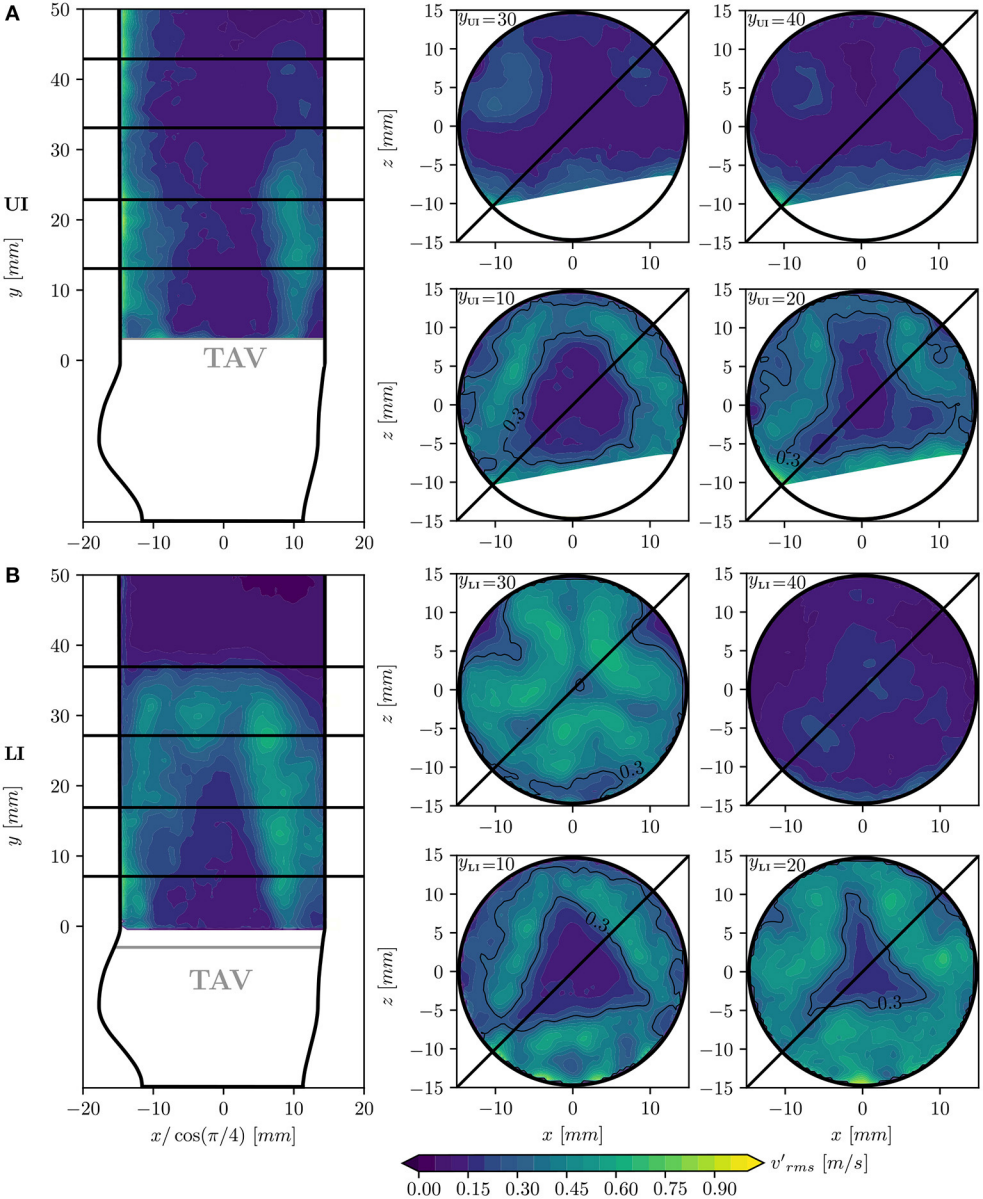
Root mean square of velocity fluctuations $v_{rms}^{\prime }$ in different cross-sections of the flow-domain: longitudinal plane at 45° together with transversal planes at *y*_LI_ = *y*_UI_ = 10, 20, 30, 40 *mm* for the UI **(A)** and for the LI **(B)**. Shear layers are highlighted by contour lines which include $v_{rms}^{\prime }$ values higher than 0.3 *m*/*s*. High $v_{rms}^{\prime }$ values were found close to the aortic wall for the UI due to measurement artifacts. This portion of the flow domain was therefore discarded for subsequent analysis.

The flow field in the aortic root was characterized by the presence of a confined forward core jet and regions of slow retrograde flow close to the aortic walls. In Figure 6, isocontour lines with ${\bar{v}}_{y}=0.5\;m/s$ show a triangular cross-section of the core jet at 10 *mm* distal to the TAV for both IH (note that in Figures 6, 7, some data in UI is not shown due to poor quality of the raw PIV data in this region). In both configurations the core jet attached to the aortic walls at *y* ≈ 25 *mm*. Compared to the LI, the core jet in the UI was wider and slower.

The retrograde flow regions are highlighted by −0.2 *m*/*s* contour lines in Figure 6 and are located between the core jet and the aortic walls in correspondence to the leaflet commissures. Between the core jet and the retrograde flow, shear layers with high velocity gradients were present.

#### 3.2.2. Velocity Fluctuations

Figure 7 shows $v_{rms}^{\prime }$ values which illustrate the turbulent nature of the flow field in the aortic root. Low $v_{rms}^{\prime }$ values characterized the flow in the core jet but high velocity fluctuations were found in the shear layers. Isocontour lines of $v_{rms}^{\prime }=0.3\;m/s$ enclose the turbulent regions with high velocity fluctuations. In the LI, the turbulent shear layer thickness increased in downstream direction, such that the potential core of the central jet closed at a distance of 30 *mm* from the TAV (*y*_LI_ = 30 *mm*). In the UI, the shear layers were less pronounced and disappeared downstream of *y*_UI_ = 25 *mm*.

#### 3.2.3. Turbulent Kinetic Energy

**Figure 8:**
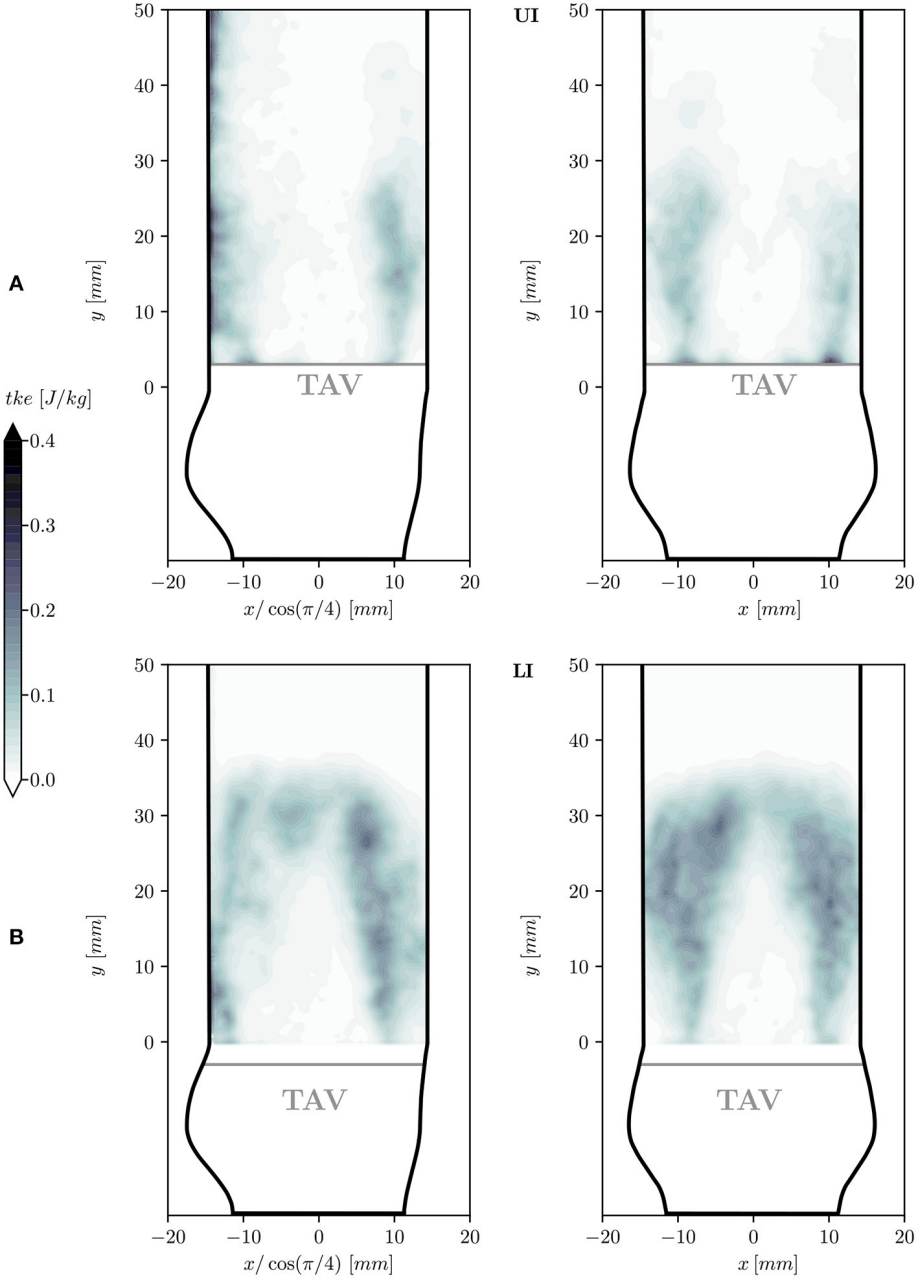
Turbulent kinetic energy density *tke* in two longitudinal planes at 45° (left) and 0° (right) for the UI **(A)** and for the LI **(B)**.

The spatial arrangement of turbulent flow described for $v_{rms}^{\prime }$ can also be appreciated from the *tke* in Figure 8. In the turbulent shear layers the energy density is in the range of 0.1 − 0.3 [*J*/*kg*] but local peak values of 0.5 [*J*/*kg*] were found. Table 2 reports the *MKE* and the *TKE* obtained by integrating the *mke* and the *tke* over the front half of the flow domain (*z* > 0) which excludes the region of low quality PIV data in the UI. For LI, *MKE* was 14% higher than in UI, and *TKE* was even 100% higher. The TI was 19.6% in the LI and 11.4% in UI.

#### 3.2.4. Turbulent Velocity Spectrum

**Figure 9:**
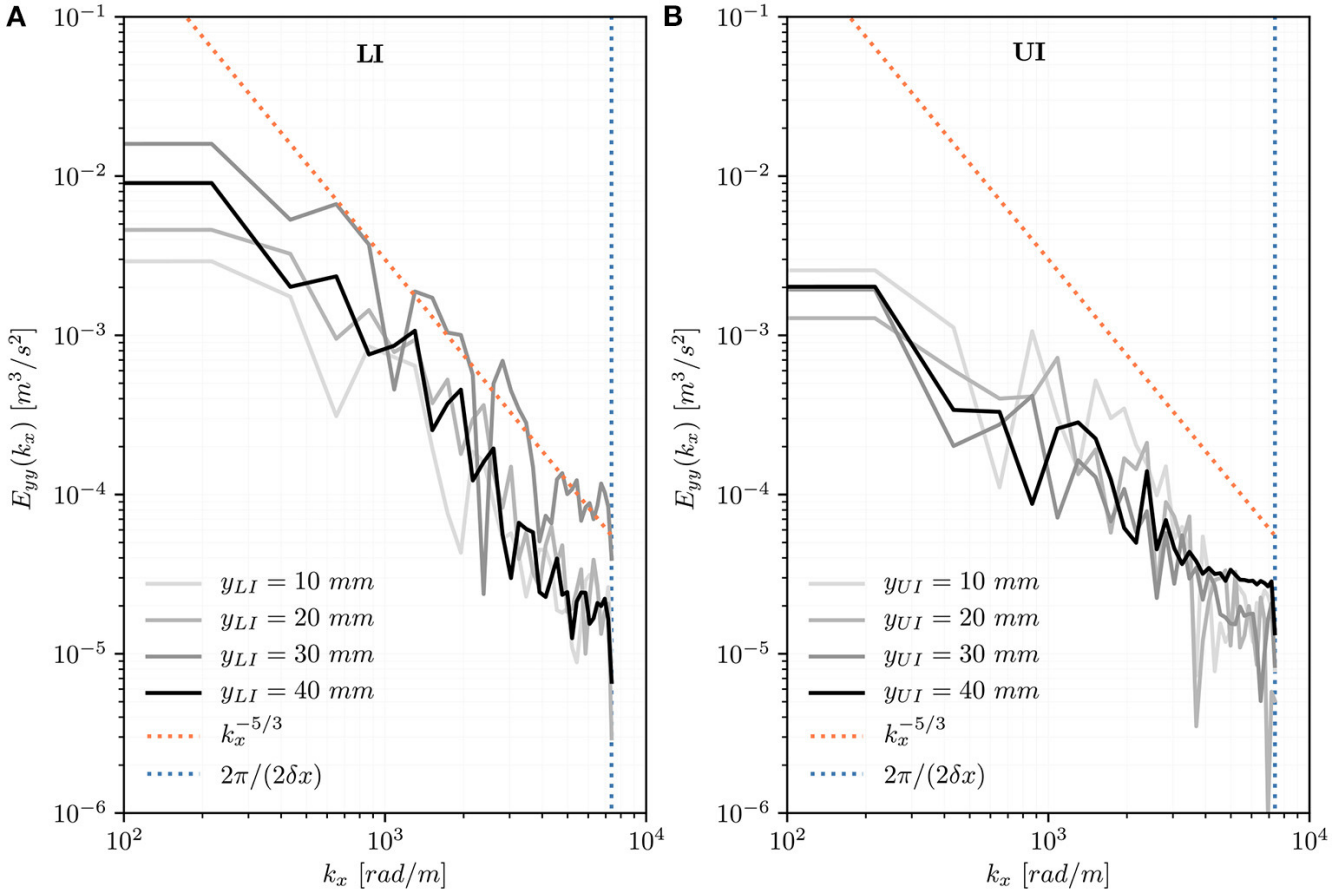
Turbulent velocity spectra *E*_*yy*_(*k*_*x*_) for points located at increasing distance from the valve (*y*_LI_ = *y*_UI_ = 10, 20, 30, 40 *mm*) on the centerline of the flow domain (*x* = *z* = 0 *mm*) for the LI **(A)** and for the UI **(B)**.

**Figure 10:**
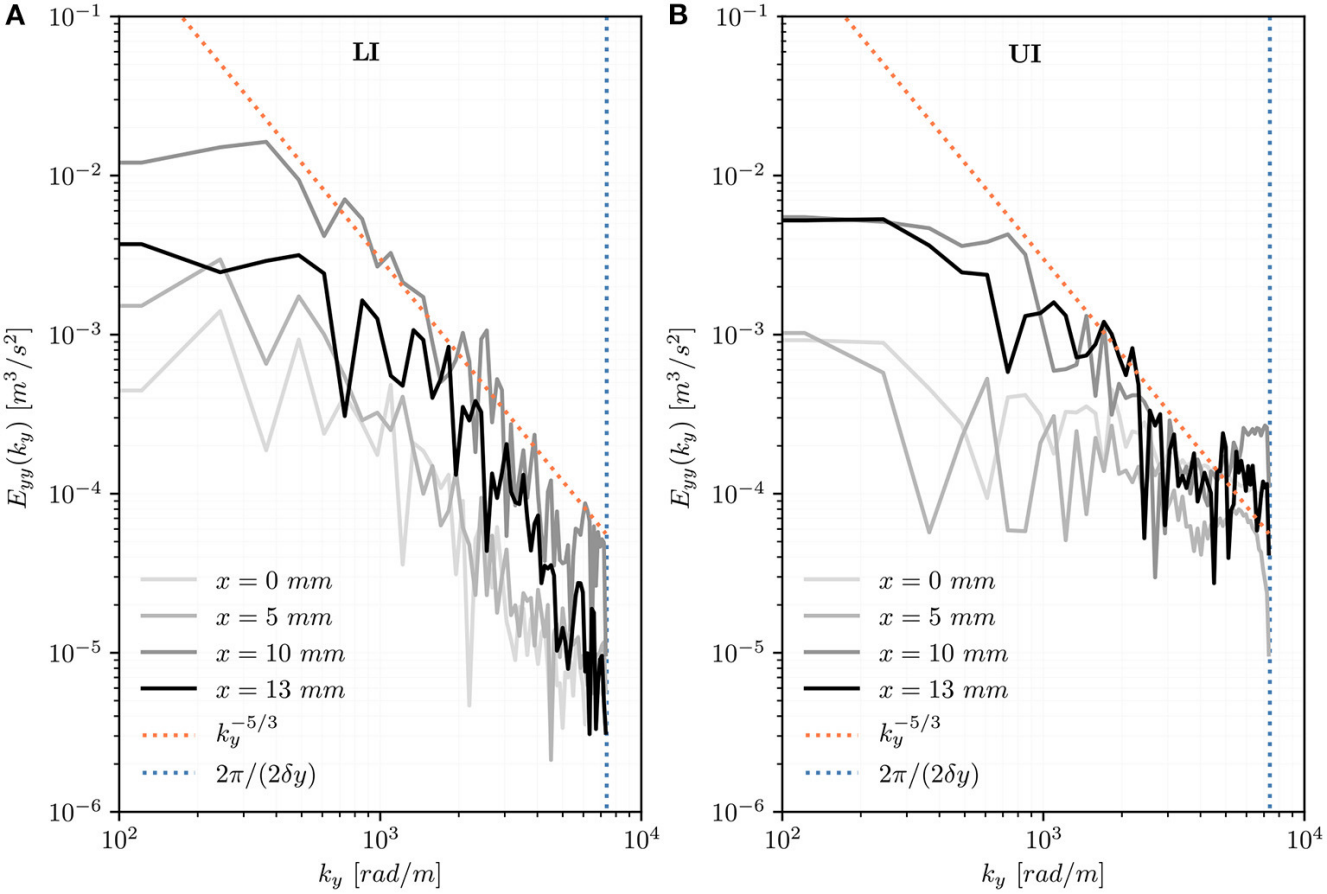
Turbulent velocity spectra *E*_*yy*_(*k*_*y*_) for points at different transversal locations (*x* = 0, 5, 10, 13 *mm*) on the center plane (*z* = 0 *mm*) at a distance of 10 *mm* from the valve's trailing edge (*y*_LI_ = *y*_UI_ = 10 *mm*) for the LI **(A)** and for the UI **(B)**.

The one-dimensional spectra *E*_*yy*_ along wavenumber *k*_*x*_ at different positions distal to the TAV (Figure 9) show the energy content of turbulent structures with different transversal length scales and are limited at large wavenumbers by the experimental resolution (*k*_*max*_ = 2π/2δ) and at small wavenumbers by the diameter of the aortic root (*k*_*min*_ = 2π/*D*_*ao*_). In the LI, the spectra show similarities with a −5/3 power-law and the spectral energy increases moving away from the TAV until *y*_LI_ = 30 *mm*. Further downstream, at *y*_LI_ = 40 *mm*, the spectral energy decays. In the UI, the spectra have a lower energy content. In Figure 10 the one-dimensional spectra *E*_*yy*_ along wavenumber *k*_*y*_ show the energy content of turbulent structures at a distance of 10 *mm* from the valve trailing edge (*y*_LI_ = *y*_UI_ = 10 *mm*) but at different transversal *x*-locations. They are limited at small wavenumbers by the length of the aortic root (*k*_*min*_ = 2π/*L*_*ao*_). In the LI, the spectral energy increases moving from the center of the flow domain toward the aortic walls. The point ***x***_*LI*_ = [10, 10, 0] *mm* is in the turbulent shear layer and the spectrum adheres to the -5/3 power-law for a wide range of wavenumbers *k*_*y*_. Closer to the wall, at ***x***_*LI*_ = [13, 10, 0] *mm*, the spectral energy decreases. In UI, the turbulent energy is generally lower and only the points close to the wall (***x***_*UI*_ = [10, 10, 0] *mm*, ***x***_*UI*_ = [13, 10, 0] *mm*) show similarity with the -5/3 power-law for a small range of wavenumbers. The peaks in turbulent energy in the large wavenumbers should be interpreted as measurement artifacts.

#### 3.2.5. Length Scales

The Kolmogorov scale η was calculated at *y*_LI_ = *y*_UI_ = 10 *mm* and *z* = 0 for *x* = 0, 5, 10, 13 *mm* (Table 3). It shows a decreasing size of the smallest turbulent structures moving from the center toward the aortic walls for both IH. The minimum values of η were found in the shear layer. The integral scales *I*_*yy*_(*l*_*y*_) estimated in the same locations show a similar trend for large turbulent structures (Table 3).

**Table 3:** Kolmogorov η and integral length scales *I*_*yy*_ estimated at 10 *mm* distance from the TAV's trailing edge at *x* = 0, 5, 10, 13 *mm* and *z* = 0 *mm* for both IH.

***x*:**	**0 *mm***	**5 *mm***	**10 *mm***	**13 *mm***
η_LI_ [μ*m*]	142	174	83	98
η_UI_ [μ*m*]	141	163	85	89
*I*_*yy*LI_ [*mm*]	11.08	9.82	3.17	3.03
*I*_*yy*UI_ [*mm*]	10.10	6.74	3.05	7.80

## 4. Discussion

### 4.1. TAV Expansion

The stopper controlled the longitudinal positioning of the TAV while the degree of expansion of the TAV was limited at the lower stent extremity by the aortic annulus and at the upper stent extremity by the aortic lumen. The interaction between the lower extremity of the TAV's stent and the aortic annulus ultimately determined the degree of expansion of the TAV. From visual inspection, the TAV's stent was not fully expanded in the 23 *mm* annulus of the aortic root phantom for neither IH. During systole, the valve presented unevenly opened leaflets while during diastole pinwheeling was observed. These characteristics suggest that the TAV was over-constrained although the size of the annulus was within the recommended range for this valve. A similar leaflet configuration was oberved by Luraghi et al. (39) after performing FSI simulation with an high fidelity CoreValve Evolute R (*Medtronic Inc, Minneapolis, Minnesota, USA*) model in a patient-specific domain including aortic root and calcifications reconstructed from computed tomography images.

Moreover, different degrees of expansion of the TAV were observed between the upper and lower implantation due to the different anchoring surfaces at the annulus. In the UI, the anchoring surface was smaller, allowing the TAV to reach a higher degree of expansion at the level of the sinus portions. This led to a larger *GOA* and *POA* during the systolic phase and a less prominent pinwheeling (*PI*) during diastole compared to the LI (Figure 5).

These differences in TAV expansion (and all the effects that follow from this) are a result of the specific configuration of this experiment with an idealized aortic root geometry. Therefore, it would not be fair to conclude that a more distal IH generally leads to a higher degree of expansion (and possibly a less turbulent flow). However, the observed differences, which were induced in this experiment by modifying the IH, could arise in reality from a variety of implantation mismatches due to the high degree of freedom of implantation procedure and due to the variability between patients aortic root geometries and degree of calcification. Moreover, an incomplete stent expansion of 10 − 15% is commonly considered acceptable in clinical practice (40). Therefore, incomplete TAV expansion with corrugated leaflets and reduced orifice area (as seen in the LI configuration) could be a common outcome of TAVI if the valve is not optimally positioned.

### 4.2. Flow Characteristics

#### 4.2.1. Turbulent Kinetic Energy

The mean systolic flow field in the aortic root was characterized by the presence of a fast antegrade central jet and a slow retrograde flow close to the aortic walls (Figure 6). This overall flow topology has already been documented for surgical bioprosthetic valves (36). The antegrade and retrograde flow domains were separated by shear layers where the velocity fluctuations were predominant and showed high levels of *tke* (Figure 8). In the LI these turbulent shear layers converged and created a more homogeneous turbulent region at *y*_LI_ = 30 *mm*. In contrast, the turbulent shear layers in the UI were less pronounced and remained confined between the core jet and the aortic walls. This was connected to higher levels of turbulence in the LI for which *TKE* was twice as high as in UI. Likewise the turbulence intensity was higher for LI (19.6%) than for UI (11.4%).

We attribute these differences to the reduced stent expansion and the more marked leaflet corrugation in LI, because it constrained the jet to a smaller opening area with higher velocities and a higher Reynolds number in the central jet. Furthermore, the corrugated shape of the leaflets trailing edge represented an obstacle for the forward motion of the fluid and likely triggered instabilities which contributed to the onset of turbulence.

The increased turbulent flow generated in the LI could potentially have a negative effect on the durability of the valve. Moreover, fluttering of the leaflets was observed from the axial high speed recordings in both implantation configurations. This fluttering was similar to one found for surgical valves in experimental and numerical studies (13, 41) and may further compromise the TAVs long-term performance (42).

Hatoum et al. (29) performed 2D particle image velocimetry measurement of an annular (*SAPIEN 3 26-mm, Edwards Lifesciences*) and a supra-annular (*Evolut 26-mm, Medtronic*) TAV in different types of silicone phantoms: the supra-annular TAV showed consistently higher levels of *tke*. Similarly to what was observed in the present study, Hatoum et al. found turbulent wakes of different intensity which impinged on the aortic walls. At peak systole, Hatoum et al. reported peak *tke* values of 0.59 *J*/*kg*, which are similar to the peak values of 0.5 *J*/*kg* found in the present study. Hatoum et al. concluded that efforts are needed to investigate the intrinsically three-dimensional nature of turbulence. The present work corroborate the findings of Hatoum et al., showing that turbulent flow develops during the systolic phase after TAVI and that turbulent wakes impinge the aortic walls, posing a risk for the endothelial cells that might be exposed to unphysiological stress loading (16).

Gülan et al. (43) tested two TAVs (*Strait Access Technologies, Cape Town, South Africa*) inside an anatomically shaped silicone phantom featuring the aortic arch. The velocity field was extracted with 3D particle tracking velocimetry (3D PTV) and a spatially averaged turbulent kinetic energy of 0.02 − 0.03 *J*/*kg* during the systolic phase was found. This corresponds well to values in the present study of 0.03 − 0.04 *J*/*kg* (UI) and 0.06 − 0.07 *J*/*kg* (LI). Differences between experimental results may be attributed to the different valves, implantation positions, aortic root geometries and dimensions of regions of interests.

Recently, Bessa et al. (44) studied with stereoscopic particle image velocimetry the effect of tilting the aortic valve in the aortic root annulus. The influence of the inlet flow orientation was assessed also by considering the spatial distribution of the *tke* in the aortic root. In their study, G.M. Bessa et al. found that the maximum values of *tke* were distributed along the inlet jet boundary as described in the present work.

Finally, we compare our results with a recent computational investigation by Manchester et al. (45) who conducted large eddy simulations (LES) in a patient specific dilated ascending aorta with aortic valve stenosis and investigated the effect of turbulence in relation with energy losses and wall shear stresses. In their study, they recognize the high velocity jet entering the dilated ascending aorta as one of the primary sources of turbulence production. Similarly to the present work, turbulent flow was concentrated in the shear layers surrounding the high velocity jet. Manchester et al. underlined that the turbulence production was further amplified by the dilated aortic root which provided space for turbulence to develop. This numerical evidence agrees with the results of the present study, where the higher level of turbulence in the LI was associated with a faster and narrower central jet which left more room for the turbulent shear layers to develop between the jet and the wall.

#### 4.2.2. Length Scales and Spectra

Quinlan and Dooley (46) pointed out that the energy spectrum of turbulence must be considered as a whole when the effect of turbulence on biological structures is investigated. In the LI, the turbulent velocity spectra *E*_*yy*_(*k*_*x*_) on the centerline showed increasing spectral energy in downstream direction until *y*_LI_ = 30 *mm* (Figure 9). In the UI, no such trend could be observed and the spectra had lower energy than in the LI. In both IH, the spectra adhered reasonably well to the -5/3 spectrum suggesting the presence of a Kolmogorov-type energy cascade. Close to the leaflets' trailing edge (*y*_LI_ = *y*_UI_ = 10 *mm*) the spectra showed a more differentiated situation (Figure 10): In the UI, the *k*^−5/3^ behavior could only be observed within the shear layers for a small window of wavenumbers. Close to the centerline, the UI spectra did not exhibit a −5/3 slope. In the LI, all spectra showed a −5/3 slope. Their energy levels and the size of the inertial ranges increased from the center (*x* = 0 *mm*) toward the shear layer (*x* = 10 *mm*). For *x* = 13 *mm*, the energy levels decreased probably due to the effect of the aortic wall. This quantitative evidence highlights the presence of well-developed turbulent flow mostly confined in the (growing) shear layers and it corroborates the earlier observation that LI leads to significantly higher levels of turbulence. The spectra agree reasonably well with the spectra presented by Becsek et al. (13) who studied a surgical bioprosthetic valve using a computational model. The quantitative agreement is best for the LI, whereas the UI shows lower energy levels than the data of Becsek et al.

The limited resolution of the experimental method can be interpreted as a low pass filter which affects the calculation of velocity gradients. Therefore, the energy dissipation might be underestimated and as a consequence the Kolmogorov scale may be overestimated. Nevertheless, the computed Kolmogorov scales (Table 3) are similar to those reported in previous experimental studies (27, 28). Lie et al. (28) conducted *in-vitro* tests on three clinically used bileaflet heart valves (*St. Jude Medical, CarboMedics and Edwards Tekna*) with 2D laser doppler anemometry. Performing the experiment at *Re* = 12, 000 and applying the Taylor frozen eddy hypothesis they obtained the smallest length scale in the range of 20 − 70 μ*m*. Li et al. (27) tested a St. Jude Medical valve with 2D particle image velocimetry and accounted for the limited resolution through a sub-grid scale model. They obtained a Kolmogorov scale of 75 μ*m* at peak flow (*Re* = 7500). It must be noted, however, that both studies focused on mechanical valves at higher Reynolds numbers. In such conditions, the aortic flow is potentially more turbulent and the resulting Kolmogorov scale smaller.

### 4.3. Limitations

As indicated by Raghav et al. (47) it is important to estimate the uncertainty related to the measured quantities. To this end, the uncertainty of the mean streamwise velocity $U_{{\bar{v}}_{y}}\left(x\right)$ and the uncertainty of the turbulent kinetic energy density *U*_*tke*_(***x***) were computed according to Sciacchitano and Wieneke (48). Spatially averaging the normalized values of uncertainty an $U_{{\bar{v}}_{y}}<1\%$ and an *U*_*tke*_ < 11% were found for both implantation configurations (see Supplementary Material).

The final TAV's configuration and stent expansion are strongly affected by the rigidity of the phantom annulus which limited the expansion of the TAV's stent. However, it must also be taken into account that during implantation, practitioners do not remove the calcified native leaflets which may introduce a similar rigid mechanical obstacle to the radial expansion of the TAV. Another limitation was the absence of the aortic arch which might have affected the impingement location of the turbulent wake in the aorta. Nevertheless, the turbulent wake was observed in the first part of the aortic root (within a distance of 2 − 3 aortic diameters from the aortic annulus) where the effect of secondary flows generated by the curvature could be considered small. Furthermore, the experiments were performed at room temperature and not at human body temperature, potentially weakening the anchoring radial forces and reducing the final expansion state of the TAV. To mitigate this effect the silicone phantom together with the TAV were immersed for 5 minutes in water at 37° before integrating them in the pulse replicator.

The different implantation positions (LI, UI) were chosen *ad hoc* and the resulting effects on TAV expansion could be considered artificial. Therefore, we would like to emphasize again that this study did not intend to evaluate the clinical outcome of different implantation positions. The two configurations were chosen to highlight that small differences in implantation positions may lead to implantation mismatches resulting in significant changes in the turbulent flow behind TAV.

Finally, the effect of the limited experimental resolution on the estimation of the Kolmogorov scale was quantified assuming isotropy and homogeneity of turbulence and resulted to be approximately 5% in the LI and 20% in the UI (see Supplementary Material).

## 5. Conclusion

The present experimental study characterized the turbulent flow behind a self-expendable TAV. The valve was implanted in a semi-rigid silicone phantom mimicking the aortic root geometry in two different supra-annular configurations: a lower implantation position (LI) and an upper implantation position (UI). The TAV was tested in an experimental apparatus including a pulse replicator and hardware for image acquisition and processing. The three-dimensional flow field was extracted in the aortic root by means of Tomo-PIV at different phases of the cardiac cycle. The valve presented similar overall flow topology but different levels of turbulence in the two different implantation configurations. In the lower implantation the TAV showed approximately 8% higher turbulent kinetic energy. In both configurations, turbulent flow was strongest in the shear layers surrounding the central jet. These turbulent shear layers impinged the aortic wall indicating that valve turbulence might play a detrimental role in the endothelial cell turnover. The turbulent energy content was presented by turbulent velocity spectra comprising well developed inertial ranges with −5/3 power-law decay. This illustrated the turbulent nature of the shear layers and the higher degree of turbulence of the TAV in the lower position. Axial views of the valve showed a smaller opening area with more corrugated leaflets during systole, as well as a more accentuated pinwheeling during diastole in the lower implantation position due to a reduced expansion of the TAV's stent in the aortic root. This suggested that the uneven opening of the leaflets and the resulting irregular orifice area amplified the onset of turbulence in the lower implantation position.

The present study highlights how implantation mismatches, which can be common in TAVI, may affect the structure and intensity of the turbulent flow in the aortic root. Leaflet corrugation during systole and pinwheeling during diastole could be considered as kinematic indicators for the presence of intensified turbulent flows that could contribute to the TAV's structural deterioration. Therefore, careful implantation planning by the heart team should also consider the patient-specific aortic root geometry and calcification degree which may lead to uneven or incomplete TAV expansion.

## Data Availability Statement

The raw data supporting the conclusions of this article will be made available by the authors, without undue reservation.

## Author Contributions

LP contributed in performing the experimental measurement, post-processing, analysis and interpretation of data, and writing and critical revision of the manuscript. SZ contributed to the post-processing, analysis of experimental data, and critical revision of the manuscript. DD contributed in the post- processing and analysis of experimental data. DH contributed in the study design and supervised the experimental campaign. DO contributed to the post-processing, analysis and interpretation of data, writing and critical revision of the manuscript, as well as study design and supervision. All authors contributed to the article and approved the submitted version.

## Conflict of Interest

The authors declare that the research was conducted in the absence of any commercial or financial relationships that could be construed as a potential conflict of interest.

## Publisher's Note

All claims expressed in this article are solely those of the authors and do not necessarily represent those of their affiliated organizations, or those of the publisher, the editors and the reviewers. Any product that may be evaluated in this article, or claim that may be made by its manufacturer, is not guaranteed or endorsed by the publisher.
