# Supplementary material for: Characterization of Turbulent Flow Behind a Transcatheter Aortic Valve in Different Implantation Positions
Source: Front Cardiovasc Med. 2022 Jan 13;8:804565. doi: 10.3389/fcvm.2021.804565 (PMC8794584; doi:10.3389/fcvm.2021.804565)
Supplement: Supplementary file 1 [file Presentation_1.pdf]

Supplementary Material

1 UNCERTAINTY CHARACTERIZATION

When characterizing the uncertainty of flow fields extracted by means of PIV methods Raghav et al. (Raghav et al., 2018) distinguish between systematic uncertainty and random uncertainty. The systematic uncertainty has been minimized during the Tomo-PIV acquisitions by following the methods described by Hasler et al. (Hasler et al., 2016), (Hasler and Obrist, 2018). The random uncertainty of the Tomo-PIV system was associated with the limited amount of realizations of the experimental protocol ($N = 64$). The random uncertainty of mean streamwise velocity $U_{\overline{v}_y}(\boldsymbol{x})$ and the random uncertainty of the turbulent kinetic energy density $U_{tke}(\boldsymbol{x})$ were computed according to Sciacchitano and Wieneke (Sciacchitano and Wieneke, 2016):

$$U_{\overline{v}_y} = \frac{\sigma_y}{\sqrt{N}} \quad [m/s] \tag{S1}$$

$$U_{tke} = \sqrt{\left(\overline{v_x'^2}\right)^2 + \left(\overline{v_y'^2}\right)^2 + \left(\overline{v_z'^2}\right)^2} \cdot \sqrt{\frac{1}{2N}} \quad [m^2/s^2] \tag{S2}$$

$U_{\overline{v}_y}(\boldsymbol{x})$ and $U_{tke}(\boldsymbol{x})$ were computed in the whole data set and normalized with the local values of $\overline{v}_y(\boldsymbol{x})$ and $tke(\boldsymbol{x})$ as follows:

$$U_{\overline{v}_y} = \frac{U_{\overline{v}_y}}{\overline{v}_y} \cdot 100 \; [\%] \tag{S3}$$

$$U_{tke} = \frac{U_{tke}}{tke} \cdot 100 \; [\%]. \tag{S4}$$

Spatially averaging the normalized values of uncertainty an $U_{\overline{v}_y} < 1$ % (0.49 % in the LI and 0.85 % in the UI) and an $U_{tke} < 11$ % (10.73 % in the LI and 10.58 % in the UI) were found for both implantation configurations.

2 UNCERTAINTY QUANTIFICATION OF THE KOLMOGOROV SCALE

The effect of the experimental resolution on the estimation of the Kolmogorov scale η was quantified as:

$$U_{\eta} = \frac{\eta(\epsilon_g) - \eta(\epsilon_s)}{\eta(\epsilon_g)} \cdot 100 \; [\%]. \tag{S5}$$

where ϵ_s is the energy dissipation rate obtained from the turbulent spectrum and ϵ_g is the energy dissipation rate obtained from the velocity gradients of the velocity fluctuations. ϵ_s and ϵ_g were computed for the axial streamwise direction assuming isotropy and homogeneity of turbulence following Zheng et al. (Zheng

et al., 2021):

$$\epsilon_s = 15\nu \int_0^\infty {k_y}^2 \; \Phi_{yy}(k_y) \; dk_y; \tag{S6}$$

$$\epsilon_g = 15\nu \overline{\left(\frac{\partial v'}{\partial y}\right)^2} \tag{S7}$$

where $\frac{\partial v'}{\partial y}$ is the 1-dimensional velocity fluctuations gradient and $\Phi_{yy}(k_y)$ is the 1-dimensional power spectral density evaluated with the *periodogram* Matlab function. The average uncertainty resulted to be around 5% in the LI and around 20% in the UI (Table 1).

$x:$	$0\;mm$	$5\;mm$	$10\;mm$	$13\;mm$
$\eta_{\mathrm{LI}}(\epsilon_s)\;[\mu m]$	132.4	113.4	83.4	96.7
$\eta_{\mathrm{LI}}(\epsilon_g)\;[\mu m]$	139.0	118.2	89.9	101.9
$\eta_{\mathrm{UI}}(\epsilon_s)\;[\mu m]$	70.8	70.13	69.4	86.0
$\eta_{\mathrm{UI}}(\epsilon_g)\;[\mu m]$	98.0	92.1	84.2	99.3.0
U_η(LI) [%]	4.7	4.1	7.2	5.1
U_η(UI) [%]	27.8	23.6	17.5	13.4

Table S1. Kolmogorov scale η estimated from ϵ_s and ϵ_g together with the uncertainty U_η at $10\;mm$ distance from the TAV's trailing edge at $x = 0, 5, 10, 13\;mm$ and $z = 0\;mm$ for both IH.

3 CONVERGENCE OF MEAN STREAMWISE VELOCITY AND ROOT MEAN SQUARE OF VELOCITY FLUCTUATIONS

The calculation of the statistical quantities such as the mean streamwise velocity $\overline{v}_y(n, \boldsymbol{x})$ and the root mean square of the velocity fluctuation $v'_{rms}(n, \boldsymbol{x})$ was based on a sample of $N = 64$ realizations of the experiment at peak systole and on a sample of 24 realizations in the other phases of the cardiac cycle. The sample size was defined as a compromise between the need of obtaining statistical relevance of the computed quantities and minimizing computational cost and data storage volume.
The sample of $N = 64$ realization used at peak systole enabled to obtain sufficient convergence of the statistical quantities. Fig. S1 and Fig. S2 show the $\overline{v}_y(n, \boldsymbol{x})$ and the $v'_{rms}(n, \boldsymbol{x})$ as a function of the number of realization for the LI and UI, respectively. In the figures values are normalized with respect to $\overline{\boldsymbol{v}}(N, \boldsymbol{x})$, $v'_{rms}(N, \boldsymbol{x})$ and refers to the same spatial locations $\boldsymbol{x}$ of the flow field where turbulent velocity spectra were computed and shown in the manuscript. A convergence region of $\pm5\%$ is highlighted in orange. It can be observed that for both implantation configuration convergence of $\overline{v}_y$ was already obtained at almost all locations after 16 realizations of the experiment. Only the points in the shear layer ($\boldsymbol{x} = [5\;mm, 10\;mm, 0\;mm]$) and close to the aortic walls ($\boldsymbol{x} = [13\;mm, 10\;mm, 0\;mm]$) required more

repetitions of the experiment due to strong velocity gradients present at those locations. v'_{rms} statistical convergence required a higher number of realizations. Nevertheless, it can be observed that after 50 realization v'_{rms} was within the $\pm 5\%$ convergence region. A sample size of $N = 64$ realizations at peak systole was selected to obtain a robust convergence of the velocity fluctuation based metrics.

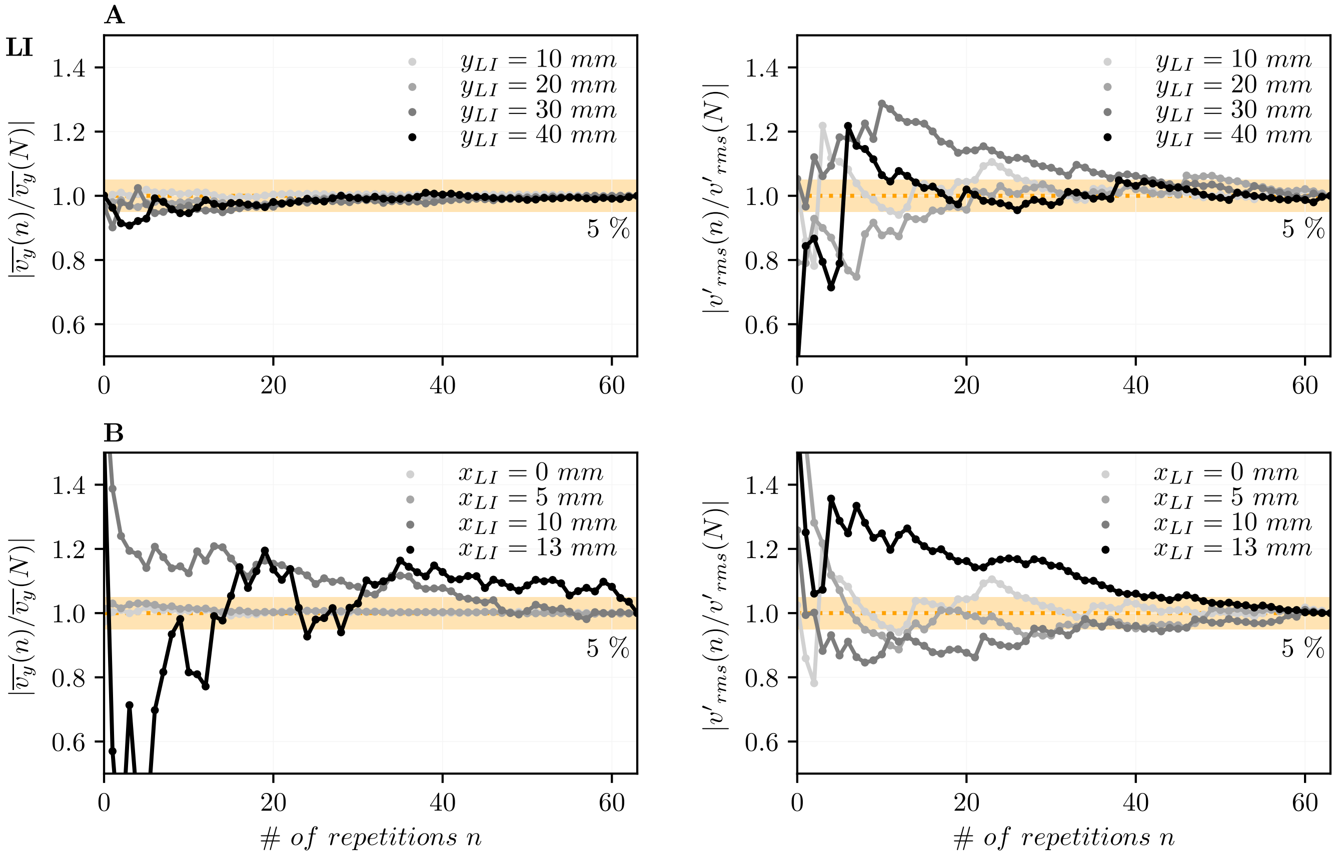

Figure S1. Normalized convergence of $\overline{v}_y(n, \boldsymbol{x})$ and of $v'_{rms}(n, \boldsymbol{x})$ as a function of repetitions number n for the LI. The convergence is reported for points located at increasing distance from the valve ($y_{\mathrm{LI}} = 10, 20, 30, 40\ mm$) on the centerline of the flow domain ($x = z = 0\ mm$) **(A)** and for points located at different transversal locations ($x = 0, 5, 10, 13\ mm$) on the center plane ($z = 0\ mm$) at a distance of $10\ mm$ from the valve's trailing edge ($y_{\mathrm{LI}} = 10\ mm$) **(B)**. A convergence region of $\pm 5\%$ is highlighted in orange.

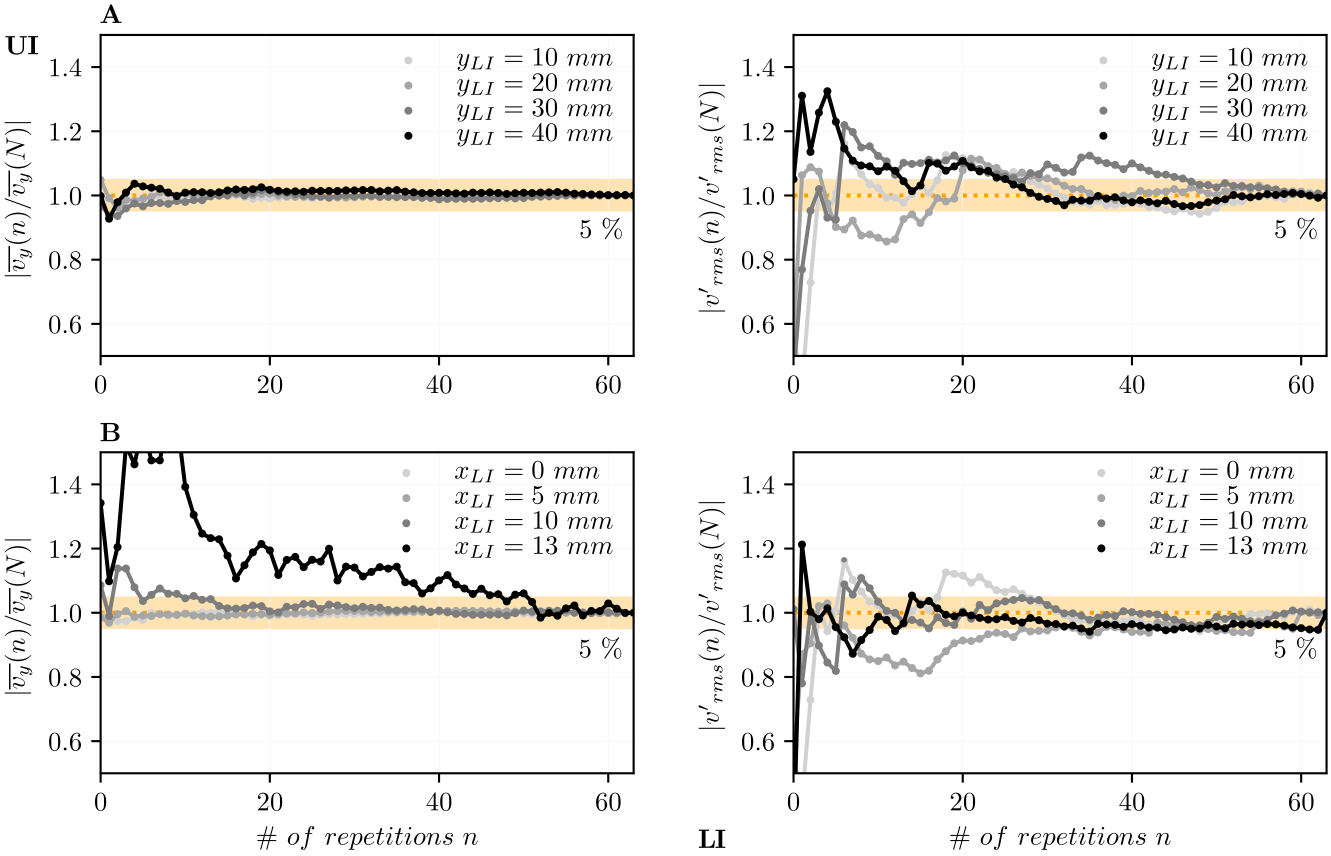

Figure S2. Normalized convergence of $\overline{v}_y(n, \boldsymbol{x})$ and of $v'_{rms}(n, \boldsymbol{x})$ as a function of repetitions number n for the UI. The convergence is reported for points located at increasing distance from the valve ($y_{\mathrm{UI}} = 10, 20, 30, 40\ mm$) on the centerline of the flow domain ($x = z = 0\ mm$) **(A)** and for points located at different transversal locations ($x = 0, 5, 10, 13\ mm$) on the center plane ($z = 0\ mm$) at a distance of $10\ mm$ from the valve's trailing edge ($y_{\mathrm{UI}} = 10\ mm$) **(B)**. A convergence region of $\pm 5\%$ is highlighted in orange.

REFERENCES

Hasler, D., Landolt, A., and Obrist, D. (2016). Tomographic PIV behind a prosthetic heart valve. *Experiments in Fluids* 57, 80

Hasler, D. and Obrist, D. (2018). Three-dimensional flow structures past a bio-prosthetic valve in an in-vitro model of the aortic root. *PloS one* 13

Raghav, V., Sastry, S., and Saikrishnan, N. (2018). Experimental assessment of flow fields associated with heart valve prostheses using particle image velocimetry (PIV): recommendations for best practices. *Cardiovascular engineering and technology* 9, 273–287

Sciacchitano, A. and Wieneke, B. (2016). PIV uncertainty propagation. *Measurement Science and Technology* 27, 084006

Zheng, S., Bruce, P., Cuvier, C., Foucaut, J.-M., Graham, J., and Vassilicos, J. (2021). Nonequilibrium dissipation scaling in large reynolds number turbulence generated by rectangular fractal grids. *Physical Review Fluids* 6, 054613
